# Chronic Subdural Hematoma Caused by Acute Myeloblastic Leukemia: A Case Report

**DOI:** 10.3389/fneur.2022.911195

**Published:** 2022-07-12

**Authors:** Guangwen Xia, Weitao Zhang, Jing Xiao, Lin Shi, Yiming Zhang, Hang Xue

**Affiliations:** ^1^Department of Neurosurgery, Yantaishan Hospital Affiliated to Binzhou Medical University, Yantai, China; ^2^Department of Neurotraumatic Surgery, The First Hospital of Jilin University, Changchun, China; ^3^Department of Hematology, Yantaishan Hospital Affiliated to Binzhou Medical University, Yantai, China; ^4^Department of Neurosurgery, The Affiliated Hospital of Changchun University of Traditional Chinese Medicine, Changchun, China; ^5^Department of Neurosurgery, Dong E Hospital Affiliated to Shandong First Medical University, Liaocheng, China

**Keywords:** acute myeloblastic leukemia, chronic subdural hematoma, mechanism, surgery, drugs

## Abstract

Chronic subdural hematoma, a common neurosurgical disease, is mostly caused by craniocerebral trauma. Chronic subdural hematoma caused by acute myeloblastic leukemia is rarely reported, and its pathogenesis and strategies for clinical treatment remain controversial. Here, we report a rare case of chronic subdural hematoma caused by acute myeloblastic leukemia. The patient's condition deteriorated quickly after admission, and emergency trepanation and drainage of the chronic subdural hematoma was performed, followed by oral administration of atorvastatin. The platelet levels continued to decrease during neurosurgical treatment. Bone marrow cytology, flow cytology, and karyotype analysis suggested acute myelocytic leukemia (AML). Then, the patient was transferred to the hematology department for chemotherapy treatment, during which there was no recurrence of hematoma. Chronic subdural hematoma caused by acute myeloblastic leukemia is a very rare disease. Surgery should be performed when the intracranial hematoma is more than 10 mm thick and the midline structures are displaced by more than 5 mm, and postoperative treatment should be supplemented with atorvastatin to prevent recurrence. Chemotherapy should be given promptly to treat leukemia after stabilization of neurological conditions.

## Introduction

Chronic subdural hematoma caused by acute myeloblastic leukemia is extremely rare, and only four cases have been reported internationally. Indications for surgery are an intracranial hematoma > 10 mm in thickness and midline structural shifts > 5 mm. Trepanation and drainage of chronic subdural hematoma is an effective surgical procedure, and postoperative oral atorvastatin administration is effective in preventing hematoma recurrence. Bone marrow cytology, flow cytology and chromosomal karyotyping may provide the basis for the diagnosis of acute myeloblastic leukemia.

## Case Presentation

**Figure 1:**
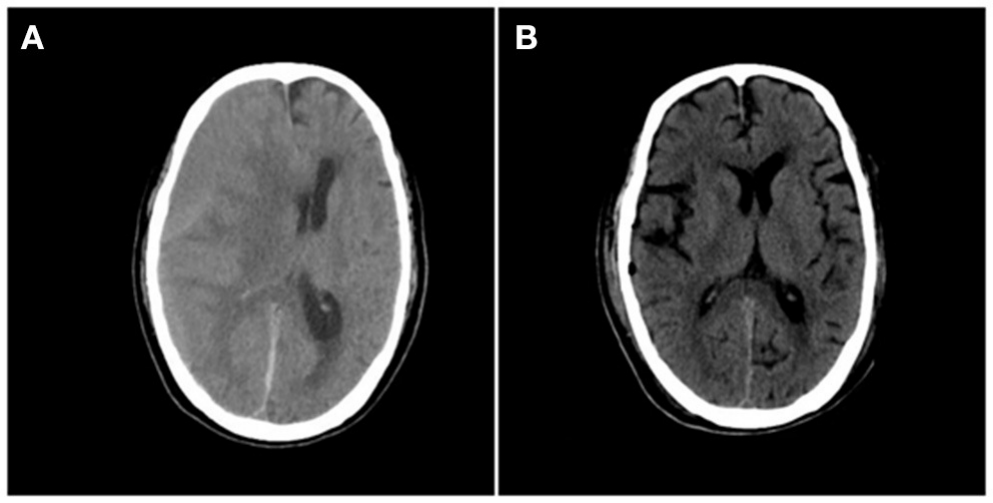
**(A)** Preoperative CT showing a crescent-shaped slightly dense shadow under the right cranial plate, with displaced midline structures and lateral ventricular compression. **(B)** Postoperative brain CT reexamination showing complete hematoma clearance without secondary bleeding.

**Figure 2:**
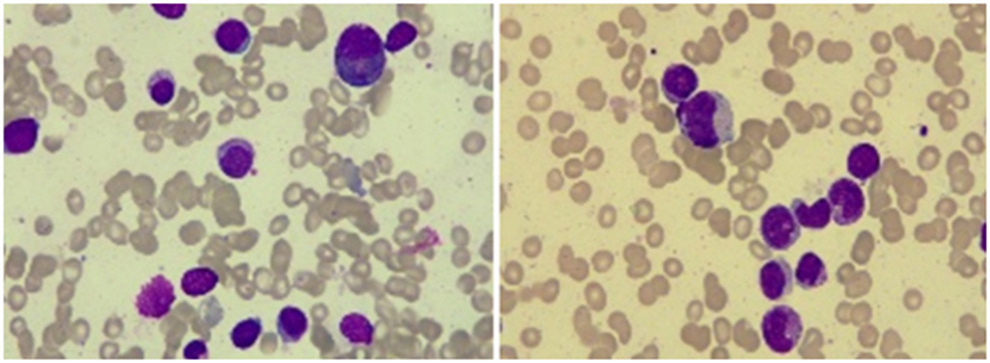
Bone marrow smear cytology showed that the proportion of monocytes increased, with promonocytes accounting for ~8.2% of these cells. The cells were variable in size and irregular, mostly with processes and varied amounts of cytoplasm. Morphological analysis of peripheral blood cells showed that the proportion of classified monocytes increased, and promonocytes were easily observed.

**Figure 3:**
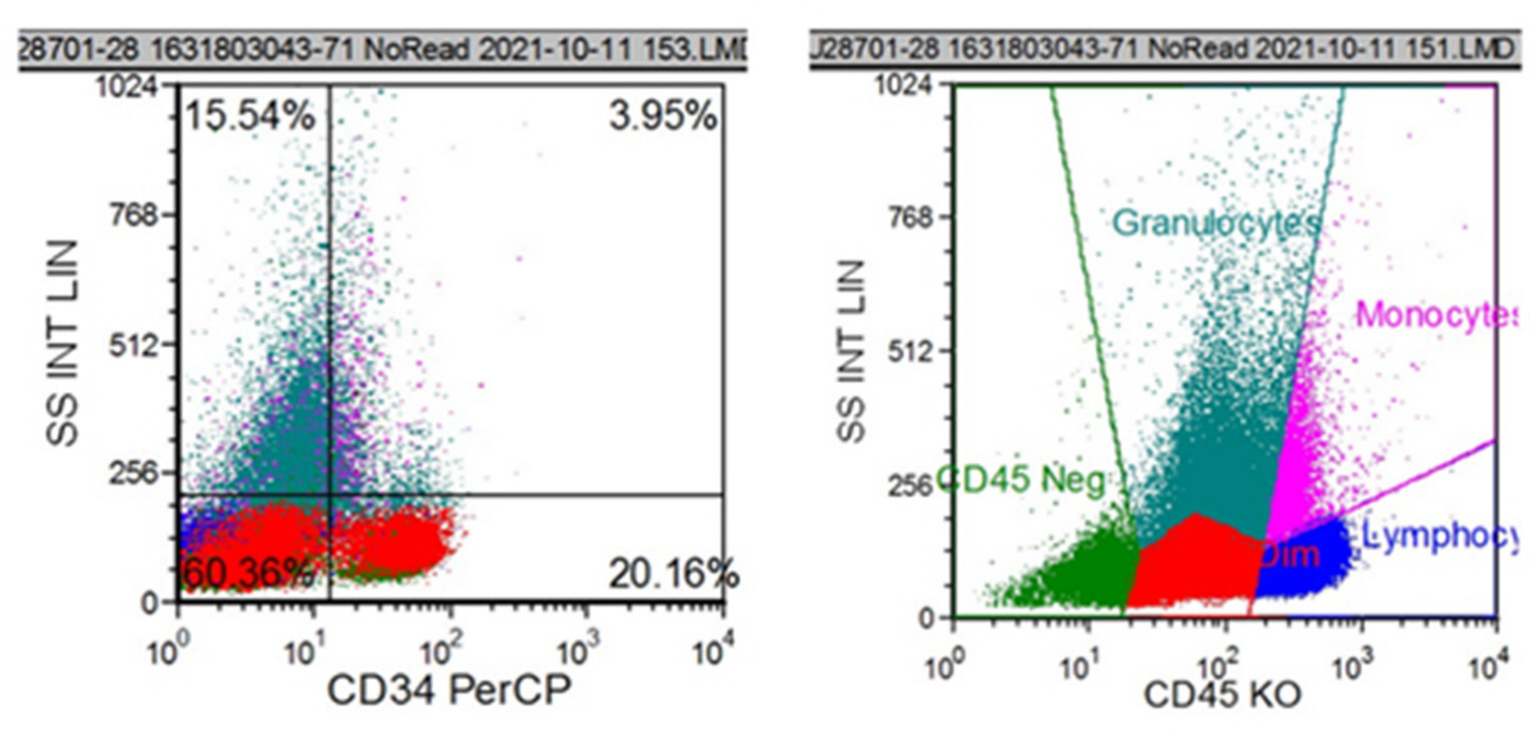
Flow cytometric analysis showed that CD34+ cells accounted for 20.16% of all nucleated cells. The immunophenotypes of these cells were CD34+, CD117+, HLA-DR+, partial 4+, partial CD11b+, and partial CD13+.

**Figure 4:**
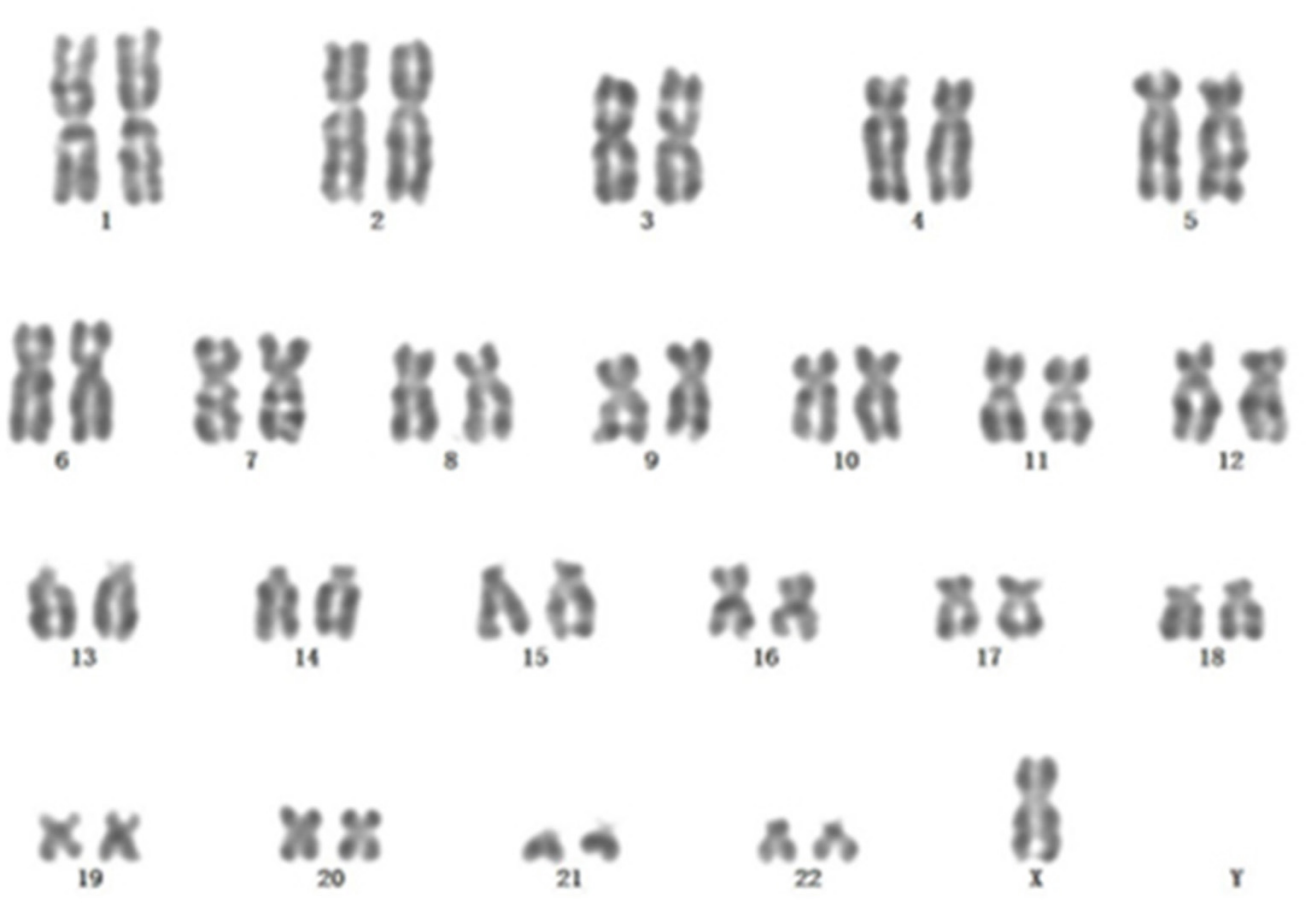
Karyotype analysis: 45, X, Y [20]. All the cells analyzed had Y chromosome loss.

A 67-year-old male patient was admitted to the Department of Neurosurgery, Yantai Mountain Hospital, Yantai City, China, on October 1st, 2021, due to a chief complaint of headache and dizziness for 1 month without apparent cause. Physical examination revealed that the patient had a clear mind and fluent speech. His pupils were 3.0 mm in diameter bilaterally, with sensitive direct and indirect light reflexes on both sides. The muscle strength of all four limbs was grade 5, with a normal muscle tone and a negative bilateral Babinski sign. He had the previous history of hypertension. Brain computed tomography (CT) suggested chronic subdural hematoma on the right frontal-temporal-parietal lobe (imaging data were lost). On admission, routine blood tests suggested a platelet count of 72 × 10^9^/L. Other laboratory tests showed no abnormalities. On the night of October 2nd, the patient's condition worsened, and his consciousness deteriorated. CT scans showed that there was a slightly higher density of crescent-shaped shadows at the right frontal-temporal-parietal lobe, the adjacent brain ventricle was compressed, and the midline structure was displaced by 15 mm (Figure 1A). Chronic subdural hematoma trepanation and drainage were performed immediately. Postoperative CT scans suggested complete clearance of the right frontal-temporal-parietal subdural hematoma (Figure 1B). The patient had intermittent fever after the surgery, and routine blood tests on October 8th showed a platelet count of 44 × 10^9^/L. Bone marrow aspiration and smear cytology (Figure 2), flow cytology (Figure 3), and karyotype analysis of bone marrow cells (Figure 4) were performed, leading to a diagnosis of acute myeloblastic leukemia. He was transferred to the hematology department for chemotherapy treatment, and intracranial hematoma recurrence did not occur during chemotherapy.

## Discussion

Chronic subdural hematoma is a common neurosurgical condition mostly seen in elderly men, with an incidence of 1–20/100,000 per year according to the literature (1). The incidence of tumor-associated subdural hematoma is 0.5–4% and is higher in patients with leukemia (2–4). According to the literature, chronic subdural hematoma caused by leukemia is most common in patients with chronic myeloblastic leukemia, whereas chronic subdural hematomas due to acute myelocytic leukemia (AML) are extremely rare, with only four cases reported thus far (Table 1). All four patients were male, two were treated surgically and two were treated conservatively with medications.

**Table 1:** Reported cases of CSDH caused by AML.

**References**	**Age (years)**	**Gender**	**WBC ( ×10^**9**^/L)**	**PLT ( ×10^**9**^/L)**	**Location of hematoma**	**Treatment**	**Outcome**
Basmaci et al. (2)	2	M	157	48	Left frontal and occipital	Craniotomy	Alicve
Comănescu et al. (3)	66	M	500	160	Left frontal and temporal	Craniotomy	Dead
Fan et al. (5)	28	M	NA	NA	Bilateral frontal and temporal	Atorvastatinan and Dexamethasone	Alive
Fan et al. (5)	68	M	NA	NA	Bilateral frontal and temporal	Atorvastatinan and Dexamethasone	Alive

*AML, acute myeloid leukemia; M, men; NA, not available; PLT, platelet count at diagnosis; WBC, white blood cell count at diagnosis*.

According to the traditional view, brain trauma causes tearing of bridging veins and consequent bleeding, resulting in the aggregation of blood in the subdural space and the induction of chronic inflammation and the formation of highly permeable neocapillaries (6). Blood continuously leaks from these immature vessels into the subarachnoid space, resulting in chronic subdural hematomas (6, 7). The above mechanism fails to comprehensively explain the cause of the formation of chronic subdural hematomas in the present case since our patient had no obvious history of head trauma or cerebrovascular diseases.

Others have suggested that dural metastases from leukemia may contribute to the development of chronic subdural hematomas in patients with leukemia. In 2020, a study of rats by Liu et al. showed that dural lymphatics are important channels for subdural hematoma drainage into the extracranial space (8). Approximately 5–8% of acute myeloblastic leukemia can present with leukocyte stasis at diagnosis (3), and leukocyte stasis can lead to lymphatic drainage dysfunction, which may block dural lymphatic drainage pathways when it invades the dura, thereby reducing the absorption of chronic subdural hematomas (8). Our patient presented with only atypical thrombocytopenia at onset and did not show leukocyte stasis; thus, this theory fails to explain the onset of the disease in this patient. In addition, leukemia cells metastasize along the walls of the blood vessels that connect the skull to the dura mater, and malignant cells are deposited in the dura mater, leading to occlusion and rupture of the dural vessels, and then blood enters the subdural space inducing chronic subdural hematomas (9, 10). In addition, the necrosis of tumor cells in the dura mater infiltrated by leukemia cells and the decrease in the number of platelets all contribute to the development of the disease (11, 12). As some scholars point out, use of anticoagulants or antiplatelet agents may predispose to subdural hematomas (5). However, our patient did not take anticoagulants and antiplatelet drugs. In conclusion, the pathogenesis of our case was that leukemic cells were deposited in the dura mater, causing occlusion and rupture of the dural vessels. In addition, the decrease of platelets also promotes the development of the disease.

The main treatment included surgical and pharmacological treatment. Surgery is required when the thickness of the hematoma exceeds 10 mm and the midline shift is >5 mm (13). Surgical options include large trauma craniotomy to remove the hematoma [now rarely used due to a high risk of trauma and postoperative complications (14)], trepanation and drainage, endoscopic removal, minimally invasive puncture to drain the hematoma, and middle meningeal artery embolization (14, 15). With long-term clinical practice, the safety and efficacy of trepanation and drainage in chronic subdural hematoma is now widely recognized, and this method is currently the most widely used procedure in clinical practice (15, 16). Our patient received CT reexamination, which showed a 15-mm shift of the midline structures and a hematoma with a maximum thickness > 10 mm; thus, trepanation and drainage were performed. Postoperative brain CT scans showed that the hematoma was completely cleared without residue.

Drug therapy can be considered if a patient is asymptomatic or has mild clinical symptoms, i.e., when the maximum diameter of the hematoma on imaging is <10 mm and the midline shift is <5 mm (13). The most widely used drugs for the treatment of chronic subdural hematoma are atorvastatin and dexamethasone. Studies have shown that subdural hematomas can disrupt endothelial cell junctions by decreasing the expression of the transcription factor KLF2, whereas statins can promote KLF2 expression and thus strengthen endothelial cell junctions. Moreover, the positive effect of statins can be greatly enhanced when combined with small doses of dexamethasone (17). Notably, even if the patient receives surgery, postoperative oral atorvastatin administration is still necessary since it reduces the recurrence rate. The patient was given atorvastatin (20 mg, orally, once daily) postoperatively, but dexamethasone was not added due to his history of hypertension (dexamethasone exacerbates hypertensive disease by causing water and sodium retention). The patient did not show any recurrence of hematoma during the chemotherapy period (7 days of chemotherapy), and there was no interruption of the chemotherapeutic effect due to surgery or drugs. The patient was followed up 1 month after surgery and recovered well with no symptoms of neurological deficit.

To conclude, chronic subdural hematoma of leukemic nature is extremely rare and has no typical clinical manifestations. However, the presence of the following manifestations often suggests the possibility of chronic subdural hematoma of leukemic nature: 1. abnormal blood test results, especially abnormal increases in white blood cell counts and decreases in platelet count and red blood cell count, as well as abnormal coagulation function; 2. chronic subdural hematoma without the history of trauma.

## Conclusions

Chronic subdural hematoma caused by acute myeloblastic leukemia is a very rare disease. Surgery should be performed when the intracranial hematoma is more than 10 mm thick and the midline structures are displaced by more than 5 mm, and postoperative treatment should be supplemented with atorvastatin to prevent recurrence. Chemotherapy should be given promptly to treat leukemia after stabilization of neurological conditions.

## Data Availability Statement

The original contributions presented in the study are included in the article/supplementary material, further inquiries can be directed to the corresponding author.

## Ethics Statement

Written informed consent was obtained from the individual(s) for the publication of any potentially identifiable images or data included in this article.

## Author Contributions

GX was responsible for writing and the figure organization. HX and GX were responsible for performing the operation. JX was responsible for collecting the bone marrow aspirate, biopsy, and examination. WZ was responsible for the pathological examination. LS was responsible for chemotherapy. YZ was responsible for the FISH examination and manuscript review. All authors contributed to the article and approved the submitted version.

## Conflict of Interest

The authors declare that the research was conducted in the absence of any commercial or financial relationships that could be construed as a potential conflict of interest. The reviewer JX declared a shared affiliation with the author WZ to the handling editor at the time of review.

## Publisher's Note

All claims expressed in this article are solely those of the authors and do not necessarily represent those of their affiliated organizations, or those of the publisher, the editors and the reviewers. Any product that may be evaluated in this article, or claim that may be made by its manufacturer, is not guaranteed or endorsed by the publisher.
